# Molecular dynamics simulations and their application to four-stranded DNA

**DOI:** 10.1016/j.ymeth.2007.02.004

**Published:** 2007-12

**Authors:** Jiří Šponer, Nad’a Špačková

**Affiliations:** Institute of Biophysics, Academy of Sciences of the Czech Republic, v.v.i., Královopolská 135, 612 65 Brno, Czech Republic

**Keywords:** Molecular dynamics simulations, Guanine quadruplex, Empirical force field, Quadruplex formation, Force field approximations, Channel cations, Quadruplex loops, i-DNA

## Abstract

This review provides a critical assessment of the advantages and limitations of modeling methods available for guanine quadruplex (G-DNA) molecules. We characterize the relations of simulations to the experimental techniques and explain the actual meaning and significance of the results. The following aspects are discussed: pair-additive approximation of the empirical force fields, sampling limitations stemming from the simulation time and accuracy of description of base stacking, H-bonding, sugar-phosphate backbone and ions by force fields. Several methodological approaches complementing the classical explicit solvent molecular dynamics simulations are commented on, including enhanced sampling methods, continuum solvent methods, free energy calculations and gas phase simulations. The successes and pitfalls of recent simulation studies of G-DNA are demonstrated on selected results, including studies of cation interactions and dynamics of G-DNA stems, studies of base substitutions (inosine, thioguanine and mixed tetrads), analysis of possible kinetic intermediates in folding pathway of a G-DNA stem and analysis of loop regions of G-DNA molecules.

## 1 Introduction

Guanine quadruplex DNA (G-DNA) molecules are considered to be the most interesting non-canonical DNA systems [1–10]. Besides experiments, G-DNA molecules were subjected to computational studies [11–30], mainly using explicit solvent molecular dynamics (MD) simulations [31–37].

MD simulation is a single-molecule atomistic *in silico* technique limited by the 1–100+ ns simulation time scale and quality of the force fields [37]. Opinions about MD simulations range from rejection of everything that is calculated to indiscriminate applications of computational methods to problems that are far beyond their applicability. Both extreme views are inappropriate. Modeling has its scope, limitations and error margins. Computers always provide numbers and structures and therefore abusing computations is quite easy. Unrealistic application of simulations provides misleading data while wisely applied simulations can rationalize the experimental results and sometimes provide important missing pieces of information not obtainable by other means. Qualitative applications of computational methods are more appropriate than quantitative studies.

When assessing the meaning of the simulation results, we should *exactly understand* what this technique does. The method deals literally with just a single molecule which has a defined *starting geometry* and subsequently undergoes 300 K *solution dynamics* on a time scale 1–100+ ns. This is a considerable difference compared with experiments that deal with assemblies of molecules and mostly under equilibration conditions. The simulations are stochastic and 10-ns-scale simulations are capable to overcome free energy barriers of ∼5–7 kcal/mol. The “computer experiment” typically samples the conformational states just around the starting geometries. The molecule does not unfold even if unfolding would occur in equilibrium solution experiments. Simulation provides detail information about the ps-scale *time development* of all aspects of the 3D structure of the single simulated molecule including exact dynamical positions of all solvent and ion molecules. There is no experimental technique that would exactly mirror this and thus a comparison of MD with experiments should not be done in a groveling manner. The most intimately related experiment is X-ray crystallography, since it provides the best starting structures, represents the best validation and also inspires computational analyses. It is vital to critically confront computations with all available experiments, keeping in mind that experiments also have error margins that can contribute to the experiment vs. theory discrepancies. On the other side, it is unfair to invalidate computational analysis only because there is no relevant experiment that could be strictly reproduced by the computations. The primary aim of making computations is not to reproduce something that is already known but to learn something new about the studied molecules. It is entirely legitimate to use computations to fill gaps in the experimental data when important features of the studied systems are not accessible by measurements. Computations can also be instrumental in providing explanations of experiments and may prevent incorrect interpretation of experiments. What, however, must be requested from computational studies is a careful consideration of the limitations, not pushing the computations beyond their accuracy limits and avoiding to oversell the results.

The task is to propose a wise set of single molecule simulations with distinct starting conditions to learn as much as possible about the studied system on the affordable time scale which typically remains below 1 μs per paper. Albeit control simulations are useful, it is not necessary to repeat exactly the same simulation over and over again. The researcher should literally play with the molecule and ask one question after another. The convergence and reproducibility of the results can be fairly well deduced from a behavior of a set of diverse simulations.

The MD method is not capable to find the correct topology of the molecule on its own. Essentially only good quality X-ray structures and in some cases NMR data provide viable starts for simulations. On the other hand, if the starting structures contain errors then MD is often capable to detect them. Force field limitations may introduce errors from negligible ones up to complete failures depending on the simulation task. Investigators with a proper expertise should be capable to fairly well assess the force field limitations while running simulations in a black-box manner is a recipe to get poor results. Sometimes the developers of computational codes contribute indirectly to the abuses, as they obviously try to provide as much method options as possible. The codes then contain options of variable applicability and reliability and inexperienced users can easily make crazy computations. The users bear the ultimate responsibility to use appropriate methods.

## 2 Methods

### 2.1 General considerations

In explicit solvent simulations, the solute molecules are immersed in a sufficiently large (extending at least 10 Å from the studied molecule) box of explicit water molecules and ions. The box is periodically extended in all directions (periodic boundary conditions). The molecules are described by simple pair-additive atomistic potentials (force fields) that treat atoms as Lennard-Jones van der Waals spheres with partial *constant* point charges localized at the individual atomic centers, linked by harmonic springs supplemented by simple valence angle and torsion profiles mimicking the covalent structure.

The pair-additive approximation means that no polarization and charge transfer effects are included, such as polarization of solute by solvent, variation of charge distributions with conformational changes, etc. Interaction of three particles is described just as a sum of three pairwise interactions while real systems are non-additive, i.e., the interaction between two particles is to a certain extent influenced by other particles (cooperativity or anticooperativity, i.e., many-body effects). The dynamical explicit solvent properly accounts for the permittivity. Compared to high-quality quantum chemical (QM) methods [38] the force field is less physically complete (and usually less accurate) but allows to study large solvated systems. This is particularly important for G-DNA where, due to the unique balance of contrasting molecular interactions, model systems smaller than complete and solvated G-DNA stems with ions have a limited significance.

### 2.2 Long-range electrostatics

Before 1995, the major limitation was the treatment of long-range electrostatic forces. In order to make simulations feasible long-range electrostatic forces must be somehow truncated. All earlier cut-off methods resulted in cumulation of brutal errors along the simulation trajectories. Such errors are particularly large for charged nucleic acids (NA) and especially for the G-DNA with its profound electrostatic interactions. This was the actual cause of a swift expulsion of the cations from the G-DNA channel and collapse of the structure reported in the first MD simulation [17]. The introduction of the particle mesh Ewald (PME) treatment of electrostatics [39,40] in 1995 eliminated this fundamental problem. The inherent periodicity of PME method could overstabilize the simulated systems [41,42] but this problem should be rather marginal compared to sampling and force field limitations.

### 2.3 Sampling limitations

The time scale of real events is much longer than the affordable simulation time scales which results in limited conformational sampling. Faster computers would improve the sampling but longer simulations may also expose force field deficiencies that have cumulative effects over time. Sampling limitation can be reduced by running multiple simulations with smart choices of starting structures or by using enhanced sampling methods (see Section 2.12). The likelihood of structural changes in simulations is nevertheless enhanced as the simulation is not fully equilibrated after its start.

### 2.4 Force field approximations

The force field is (and will likely remain in the near future for a variety of reasons) so simple that it cannot capture accurately all energy contributions simultaneously, especially when using multipurpose biomolecular force fields. Tuning the force field to reproduce a given feature can increase errors elsewhere. For example, polar hydrogens have small apparent radii when interacting with polar groups but quite large ones when they contact non-polar groups, so the fixed radius in a simulation is a compromise. The force field atomic radii are anyway fictive adjustable parameters optimized to achieve a meaningful overall force field performance.

### 2.5 Base stacking, pairing and atomic charges

The AMBER force field [43] provides a balanced description of base stacking and base pairing [44–46]. Atomic charge distributions are necessarily arbitrary, as there is no quantum mechanical operator for atomic charges [38]. There is no experiment (even a hypothetical one) to determine atomic charges. Partial atomic charges (even those that would be derived from experimental electron densities) do not correspond to any real physicochemical quantity. Thus, charge distributions can be greatly manipulated by changing the way they are derived. This should be considered when assessing molecular interactions based on the individual atomic charges, as there obviously are no ultimate “correct” charges. (So called Mulliken population analysis shows huge method and basis set dependence and should never be used.) It makes no sense to compare individual atomic charges in different force fields. What matters is how the whole set of charges performs in interaction energy calculations. AMBER derives the charges to reproduce the electrostatic potential (ESP) around the molecules [47,48]. ESP is a measurable (real) quantity which in addition determines the electrostatic part of the biomolecular interactions. No out-of-plane charges are needed for nucleobases and the van der Waals term is also well balanced.

### 2.6 Non-planarity of amino groups

Amino groups of NA bases tend to be non-planar due to a partial sp^3^ hybridization [38,49,50]. Non-planar amino groups affect stabilization of bifurcated H-bonds, close amino group contacts, non-planar G/A base pairs and some other specific interactions [38]. The force fields, in contrast, assume purely sp^2^ amino nitrogen. This should be sufficient for most interactions, as primary H-bonds stabilize the sp^2^ electronic structure. Parametrization of a force field that flexibly modulates the amino group nitrogen hybridization (including its lone pair in amino-acceptor interactions) in response to the environment would not be straightforward.

### 2.7 Description of the backbone

The sugar-phosphate backbone is considerably more difficult to deal with for two reasons: (i) it is flexible and thus the *constant* point charges do not reproduce the electrostatic potential equally well for distinct backbone substates (geometry-dependent charges would be needed, and most likely it would be desirable to go beyond the approximation of atom-centered charges) [47,48,51], (ii) it is a highly polarizable anion, with a complicated electronic structure that changes with solvation and conformational dynamics. These contributions are neglected by the non-polarizable atom–atom pair-additive force fields.

Recent studies [52–54] detected a stunning failure in longer (10+ ns) AMBER MD simulations of B-DNA using both parm94 [43] and parm99 [55] (two slightly different variants of the AMBER force field). There has been an accumulation of irreversible g+/t and g−/t α/γ backbone substates (instead of the canonical g−/g+ substate) followed by severe distorsions of the double helix. When using simple force field we rely on a mutual compensation of errors of the individual force field terms and this B-DNA degradation is a textbook example what happens when the compensation fails in a certain application. Fortunately, the α/γ B-DNA degradation can be considered as an extreme problem and for most applications the simulations are not biased to such extent.

A viable correction of the α/γ DNA backbone profile (parmbsc0) [56] was recently introduced.

The α/γ imbalance contributed to poor G-DNA loop behavior (see Section 3.5) [16]. Our preliminary data suggest that with parmbsc0 the loop description is visibly improved but not fully corrected. The imbalances in description of G-DNA loops are caused not only by the tuning of the α/γ torsional terms in parm94/99 but also by other problems which remain to be tackled. Modeling of single-stranded NA regions is one of the most challenging problems where force field errors are quite likely to be exposed [37].

The G-DNA stem simulations are not markedly affected by the α/γ problem and thus the older results remain valid. Similarly, the α/γ imbalance does not disturb RNA simulations and the overall RNA performance of the AMBER parm94/99 force field is amazing [37,57,58]. For DNA, the parmbsc0 is strongly preferred although it perhaps may introduce a small bias towards canonical backbone topologies.

### 2.8 Cations and anions

**Fig. 1:**
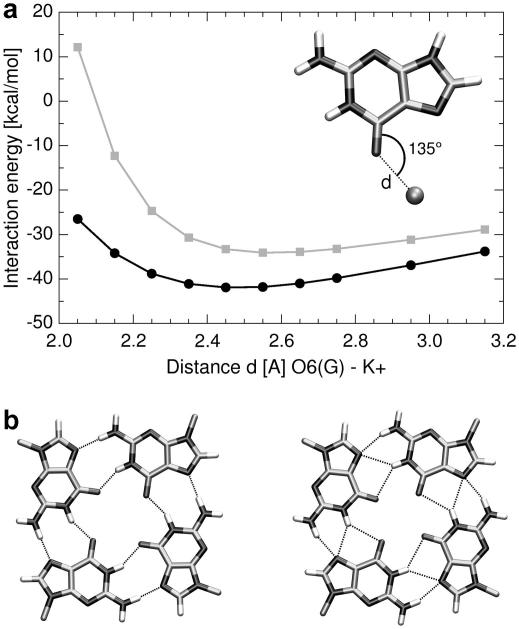
Limited accuracy of a non-polarizable force field for cation–solute interactions. (a) Dependence of the interaction energy between O6(G) and K^+^ in a G-DNA-like geometry. Force field (radius 2.6580 Å and well depth 0.000328 kcal/mol) and reference QM data are in gray and black, respectively [37]. (b) Inner guanine quartet geometries in the parallel G-DNA–X-ray geometry (left) and bifurcated geometry often seen in MD simulations [11] (right).

Performance of force fields for monovalent cations is reasonable and they sample quite well already in 25+ ns simulations [52,59,60]. Simulations well capture the overall effect of the channel ions on the G-DNA stabilization. However, both standard K^+^ and Na^+^ ions are little oversized (they look little too large). For Na^+^ the in-plane (tetrad) positions are considerably under-populated and K^+^ can even pop out of the channel. Reduction of the monovalent ion radii considerably improves the sampling inside the channel [11,12] albeit it may bias for example ion solvation energies which could affect free energy calculations. The direct binding strength between the ion and the O6 atom of guanine is underestimated by several kcal/mol when compared to QM calculations [11,14] (Fig. 1a). Simulations with Na^+^ often give bifurcated topology of some G-quartets (Fig. 1b) which likely is another consequence of the lack of polarization [11]. The force field description of the Na^+^ vs. K^+^ difference may be imperfect and studies aimed to capture the Na^+^ vs. K^+^ difference should be done with a care. We were not able to reproduce experimental [61,62] monovalent cation binding sites at the G-DNA stem-loop junctions [16].

Very deficient is the description of divalent cations associated with large polarization and charge transfer. The Mg^2+^⋯N7(guanine) interaction energy term is ∼210 kcal/mol, while the force field accounts for only ∼150 kcal/mol. Non-additivity in the first ligand shell of Mg^2+^ is ∼70 kcal/mol, mainly due to inter-ligand polarization repulsion [63]. In reality, the first-shell water molecules are heavily polarized by the ion and their H-bonding properties are very different from those of bulk water molecules [64]. The force fields can be biased towards direct (inner-shell) binding of Mg^2+^ to solute. The sampling of divalent ions is entirely insufficient in affordable simulations. In most cases it is wise to avoid divalents in simulations.

Anions have electrons localized far from their atomic centers and are thus highly polarizable, requiring polarizable force fields [65]. Formation of artifact KCl clusters with standard AMBER force field parameters was reported and such high-salt simulations may possibly expose some artifacts due to periodicity of the PME method [66]. Thus, the common approach of running simulations containing just neutralizing monovalent cations (∼0.2 M) is rather justified.

### 2.9 Protein force fields

Simulations of protein–DNA complexes require that protein and DNA force fields are mutually consistent. The latest version of AMBER protein force field is parm99SB which is considerably improved over parm99 [67]. Another recent protein force field is ff03 but it has no NA counterpart [68].

### 2.10 Ligand force fields and non-standard residues

The DNA force fields emerged after years of a careful development and testing. Thus, a major attention should be paid to make any drug force field [69]. This requires parametrization using high quality QM potential energy surfaces and testing. Many ligands are complex molecules and many of them also are non-neutral. Troubling are flexible parts of the ligands and the associated torsional profiles. Most authors prefer, however, to get their ligand force fields without much investment. One common approach is to create the force field “on analogy with the existing parameters”, which is an *ad hoc* parametrization. AMBER code allows an automated derivation of the force fields. Atomic charges are obtained consistently with the rest of the force field while the remaining parameters are assigned using GAFF (generalized AMBER force field) method [70]. However, GAFF is still approximate and the resulting force field is advised to be verified and tuned, using QM calculations and test dynamical runs. Simple ligand force fields could be very useful for some qualitative tasks [23] but are not suitable for any quantitative modeling or free energy calculations. Equally difficult would be to properly modify the backbone. Modified nucleobases (thioguanine and inosine) are easy to parametrize since the bases are planar rigid systems without torsional flexibility.

### 2.11 CHARMM and other force fields

Another major force field is CHARMM [71,72], which has basically the same functional form but a different philosophy of derivation. CHARMM has not yet been used for four-stranded DNAs while its performance for B-DNA is very good. Instability of CHARMM27 trajectories for folded RNAs has been reported [73]. The recent GROMOS force field [74] was not yet independently tested for DNA. One should avoid using force fields that were not specifically parametrized for nucleic acids. It is unlikely that these would incidentally work for DNA.

New generation of force fields should explicitly consider polarization and will be more physically complete [75–77]. Polarization force fields are successfully used, e.g., for ion solvation studies [78,79]. However, to parametrize a biomolecular polarization force field for NA is a tremendous task. It is not possible to simply add polarization terms to the existing non-polarizable force fields and the whole parametrization should start from the scratch. It remains to be seen when such force fields will be available and it is not *a priori* guaranteed that they will achieve a considerably improved accuracy. It might be quite difficult to achieve the desirable balance of all the individual terms and the appropriate cancellation of errors. Note that even such force field would not capture all non-additive effects (e.g., charge transfer associated with divalent cations) [80].

### 2.12 Enhanced sampling methods

Explicit solvent MD simulations can be combined with enhanced sampling techniques: locally enhanced sampling (LES), replica exchange (RE) and targeted MD. All these methods introduce additional approximations.

LES [81,82] splits a selected part of the molecule (e.g., a loop) into N (3–5) copies that move independently while the rest of the molecule is simulated in the standard manner. The height of conformational free energy barriers between distinct substates is reduced roughly by 1/N. LES was applied to studies of the single-stranded loops of G-DNA [16,20]. In REMD [83,84] several non-interacting copies (replicas) are simulated independently at different temperatures (e.g., 300–500 K). In contrast to LES, each replica is a copy of the whole system in standard REMD. Conformations of the individual simulated systems are exchanged using Metropolis-like formulae considering probability of sampling of each conformation at the alternate temperature.

Targeted simulation forces the sampled structure to be at a given RMSd from some reference geometry by a harmonic penalty [85]. By changing smoothly the target RMSd one can approach or separate the molecule from reference geometries. Major caution is necessary in case of complex conformational transitions as such transitions will be typically outside applicability of the method (despite that low RMSd is enforced the transitions and structures can be entirely senseless) [86].

Elevating the temperature (to, e.g., 400 K) is another crude way to overcome barriers. However, the pair-additive potential is tuned for room temperature simulations and adverse effects such as empty cavities in the bulk solvent may occur.

### 2.13 Continuum solvent methods

Costly explicit solvent calculations can be replaced by continuum (implicit) solvent methods [87–90] using Poisson–Boltzmann (PB) or more approximate generalized Born (GB) approaches, together with a surface area (SA) term which is usually obtained by scaling the solvent accessible surface area by an appropriate surface tension. These methods allow assessment of hydration and free energies that cannot be derived from explicit solvent models, thus providing relation between structures and energies. Unfortunately, these approaches introduce additional substantial approximations, require parameterization, and their results are very sensitive to parameters such as the atomic radii used to define the solute cavity. The GB/SA approach can be used to run continuum solvent MD simulations, but the range of applicability of GB/SA MD has yet to be established [91]. It shows faster sampling because of the absence of friction between solute and solvent, but the method may also significantly over-stabilize folded structures. There is no reason to use GB/SA dynamics when the computational task can be achieved by explicit solvent simulation.

Another approach is to perform standard explicit solvent MD simulations and subsequently apply the continuum solvent method (post-processing of the trajectory), by removing the explicit solvent and periodicity and averaging the energies over a sufficient number of snapshots (static frames) [89,90]. This is known as MM-GBSA or MM-PBSA free energy methods. As free energy is a state function, one can evaluate free energy differences between distinct substates without simulating the transition. MM-PBSA calculations of G-DNA require explicit inclusion of the channel ions and were instructive in comparing different substates of G-DNA stem [15]. MM-PBSA failed to predict the absolute values of DAPI binding to B-DNA [69]. For a recent assessment of the technique see [92]. To estimate the ligand-binding energies one can use either single or multiple trajectory approaches [89]. In the multiple trajectory approach, the free energy difference is evaluated using three separate simulations (trajectories) of the complex, receptor and ligand. In the single trajectory approach all data are derived from the single trajectory of the complex, i.e., it is assumed that the free energy (conformational dynamics) of the ligand and receptor would be identical in the simulation of the complex and in the separate ligand/receptor simulations. Thus any induced fit effect is neglected. However, the single trajectory approach may be more robust by canceling sampling errors in the intramolecular terms. These errors may be very significant when the sampling of solute substates in the separate trajectories is mutually inconsistent, which is a likely scenario [69]. Explicit inclusion of a subset of water molecules around the binding site could improve the binding free energy predictions [69].

In summary, continuum solvent approaches can provide useful insights into the molecular interactions but accuracy of such calculations is rather modest. An order of magnitude change of the ligand-binding equilibrium constant corresponds to just ∼1.4 kcal/mol free energy difference at 300 K. Quantitative accuracy is not achievable with the contemporary computational methods unless the method is specifically trained for a given system using known experimental data.

### 2.14 Free energy perturbation, thermodynamics integration, potential of mean force and umbrella sampling

**Fig. 2:**
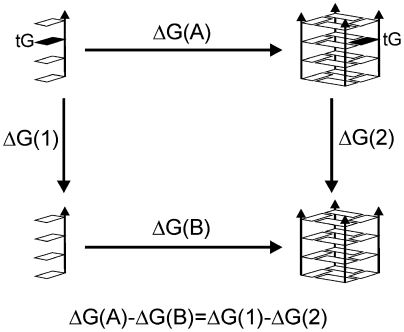
Thermodynamic cycle for guanine to thioguanine (tG) substitution in a G-DNA stem [13]. The vertical processes Δ*G*(1) and Δ*G*(2) are those computed by TI method.

Free energy perturbation (FEP) and thermodynamic integration (TI) are capable to determine the free energy difference (Δ*G*) between two states (characterized by values of a perturbation parameter 0 and 1) in the course of explicit solvent simulations. ΔG is calculated as a step by step transition connecting states 0 and 1. For example, guanine can be “mutated” to thioguanine. Although this is an unphysical transition process it is legitimate in computations as free energy is a state function and one can use thermodynamics cycle (Fig. 2). TI was used to study free energy changes due to guanine to 6-thioguanine substitution in duplexes, triplexes and G-DNA with a good agreement with available experiments [13]. An overview of free energy calculation methods is available in [93,94]. FEP and TI are more accurate compared with the continuum solvent methods. However, the simulated changes and transitions must respect the limits of the available sampling. Thus, purine to pyrimidine substitution is a manageable albeit not easy task for TI. Inserting an intercalator into a duplex DNA is beyond the applicability limit.

The change in free energy between the initial and final states can be expressed as a function of a spatial coordinate (i.e., an internal coordinate of the system or a coordinate in Cartesian space). Simply saying, the change in free energy corresponds to the average force acting along the chosen coordinate and is thus referred to as a potential of mean force (PMF). PMF calculations are useful for studying conformational changes, molecular associations and chemical reactions. The standard method to determine PMF along the coordinate is the umbrella sampling method [95], which applies an artificial biasing potential to the system to extend the range of sampling that may not be explored extensively via regular MD simulations. Umbrella sampling simulations were applied to studies of base pair opening in B-DNA albeit the results appear to be force field dependent [96,97].

### 2.15 QM calculations on tetrads

*Ab initio* QM studies of nucleobase quartets [98–100] are more physically complete than force field calculations. They, however, dissect exclusively the intra-tetrad (or partly inter-tetrad) interactions and neglect the interplay of forces in G-DNA which mainly stems from long-range electrostatics and solvation. Note that while G-quartets without ions are stable in gas phase they are not stable in solution. Meyer and co-workers found that a quartet stack sandwiching the Na^+^ with square antiprismatic coordination was by almost 10 kcal/mol more stable than a cubic coordination [101]. While the calculations likely nicely capture the correct trend the calculated energy difference is enormous, which may be due to the *in vacuo* environment or some other limitations (see below).

QM calculations of single quartets are straightforward. There are, however, numerous problems when calculating two quartets simultaneously even when attempting just single point energy estimates. The usual DFT (density functional theory) methods are very good for H-bonding and cation–base interactions, superior to force fields. However, DFT notoriously fails for stacking, thus the vertical interactions between guanines cannot be described in a realistic way. For stacking, AMBER force field is superior to medium cost QM methods. DFT is typically neglecting the dispersion energy entirely. Accurate calculations of stacking require MP2 method with large basis set of atomic orbitals or even expansion to the basis set limit, further followed by exceptionally costly coupled cluster correction which scales computer requirements with ca. sixth power of the number of atoms included. The largest system for which such data is available is guanine dimer [44]. DFT methods augmented by empirical dispersion term [102] could in future be used for stacked G-quartets.

Gradient optimization of a two-quartet structure would be further affected by two additional uncertainties. The first problem is the mathematical artifact known as the basis set superposition error (BSSE). BSSE originates in the incompleteness of the basis set of atomic orbitals and causes an artefactual stabilization of molecular complexes. It can be easily corrected for single-point calculations on fixed geometries but remains uncorrected in the course of optimizations. Although BSSE could compensate for the missing dispersion in DFT calculations the degree of the compensations of these two big errors is uncertain. The second problem is the lack of the environment that certainly would exert a non-negligible effect on the system. For further details about the *ab initio* QM methodology see [103,104].

### 2.16 Gas phase simulations

Recent studies [27–29] indicate that many cation-stabilized G-DNA structures may survive with only modest perturbations up to 1 μs gas phase (*in vacuo*) simulations [27]. Such simulations are aimed to complement mass spectrometry experiments. A possible weakness is that the AMBER force field has been designed for condensed phase studies. Further, and more importantly, in the course of electrospray ionization mass spectrometry (ESI-MS) experiment, the DNA molecules are transferred to the gas phase with an unnatural charge of −3 to −8e. This charge should be somehow distributed along the molecules while its exact spread or localization is unknown. When deviating from the natural charge of −1 per phosphate the electronic structure of the DNA changes and this would require an overall reparametrization. The lack of any structural experimental data precludes further assessment of such simulations and one should be very careful to not over-interpret these otherwise interesting results. Evaluation based on cross sections might be of a limited value for something as spherical as G-DNA [27]. Note also that the actual process of taking the molecule from solution to the gas phase is not modeled and thus the starting structures taken, e.g., from X-ray may be non-relevant. Actually, one of these studies [105] suggested quadruplex stems to be present in solution without the stabilizing ions, i.e., that the ions are not needed for G-DNA structure to survive. This speculation contradicts solution and X-ray experiments. Further, solution simulations unambiguously show that a vacant G-DNA stem is a ns-scale unstable structure and can be ruled out in solution [11,15]. Vacant stem either captures swiftly ions from the bulk solvent or collapses entirely. We see no force that could prevent the ions to jump into a vacant stem.

## 3 Representative MD simulations and their results

### 3.1 Simulations of G-DNA stem

**Fig. 3:**
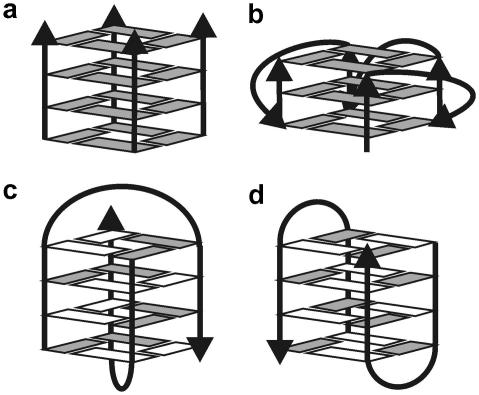
Guanine quadruplex structures: (a) parallel without loops, (b) parallel with groove loops, (c) and (d) antiparallel with diagonal and lateral loops, respectively. Open and gray boxes indicate syn and anti conformation of guanine residues.

**Fig. 4:**
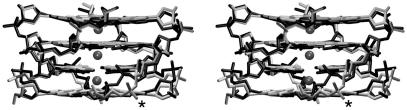
Stereo overlay of the final averaged simulated structure [11] (gray) and the initial X-ray coordinates (black). A phosphate group adopting a bistable conformation is indicated by an asterisk.

MD simulations of parallel d(G_4_)_4_ stem (Fig. 3a) [11] starting from the 0.95 Å resolution X-ray structure [5] nicely reproduced (kept stable) all its basic structural features, including bistability of the phosphate groups (Fig. 4). The high-resolution X-ray structure, however, allowed to discern local imbalances in the quartet–cation interactions (see Section 2.8). RMSd between the X-ray and simulated structures was below 1 Å, less than in common NA simulations. Thus, simulations show that G-DNA stem is exceptionally rigid. Note, nevertheless, that RMSd is an uninformative metric for MD studies for a variety of reasons. RMSd between simulated and X-ray structure is larger than between two X-ray structures since for example standard bond lengths and angles subtly differ from the X-ray dictionaries. Larger simulated NA molecules with RMSd 2–5+ Å can still well agree with the target structures, as judged by inspection of H-bonds, stacks etc. In addition, one can evaluate either RMSd for *averaged MD structure* or time development of *instantaneous RMSd* along the trajectory. For intrinsically dynamical molecules the instantaneous RMSd may be quite large while RMSd of the averaged structure may still drop to 1–2 Å. Simulations provided stable trajectories for the diagonal loop NMR structure of d(G_4_T_4_G_4_)_2_ [4] (Fig. 3c) while the lateral (edge) loop variant based on X-ray structure [106] (Fig. 3d) resulted in substantial initial relaxation of both the stem and loops, indicating errors in the experimental structure.

MD simulations were applied to studies of complexes between G-DNA and ligands (including, e.g., anthraquinones, acridines and porphyrins) to complement various experimental data lacking atomic resolution (i.e., diffraction experiments, gel mobility shifts, FRET, etc.) [22–24,107] (see cautionary comments in Section 2.10). Possible arrangements for the G-DNA–ligand complex include two basic binding modes: an external binding, when ligand is stacked at the end of the quadruplex stem, and an intercalated binding, when ligand is sandwiched between two adjacent quartets. Several studies [22,23,107] revealed the preference for the external binding mode over the intercalation one. External binding does not require conformational changes in the quadruplex stem and is also consistent with the relatively rapid kinetics observed for the complex [23]. Very crude free energy estimates from MD simulations of G-DNA–porphyrin complexes [22] indicate that Δ*G* for external binding mode is driven by an enthalpy term Δ*H* (with a small unfavorable entropy term *T*Δ*S*) while Δ*G* for intercalation is driven by a large *T*Δ*S* (complemented by a small Δ*H*).

### 3.2 How many cations are needed in G-DNA stem?

**Fig. 5:**
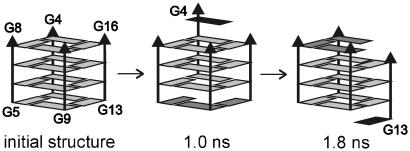
Vertical strand slippage observed in simulation of a vacant stem [11]. Strands are labeled A–D and guanines are shown as boxes.

The parallel four-tetrad quadruplex remains entirely stable with two cations in the channel, confirming that the stem is capable to achieve smooth equilibrium exchange of cations with the bulk solvent, experimentally observed on the hundreds of microseconds to millisecond time scale for the central ions [108]. The rigidity of the quadruplex is immediately lost when ions are absent in the channel [11,19,30]. Waters quite swiftly move into the channel but are not able to sufficiently stabilize the stem. Rearrangements involving vertical slippage of one strand (“strand walking”) were reported for four-quartet stem leading to temporary formation of a “slipped” molecule (Fig. 5). This slipped stem can be stabilized by placing cations back into the channel, leading to a stable cation-stabilized slipped quadruplex. MD simulations of longer vacant stems (containing up to 24 quartets) are more stable and indicate that increased number of quartets improves the stability of the G-DNA structure [30].

**Fig. 6:**
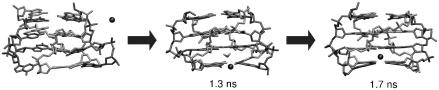
Spontaneous interception of a bulk cation by the initially vacant stem [12]. The middle figure shows the ion waiting at the channel entrance while the channel cavity is occupied by the water molecule.

The vacant quadruplex spontaneously intercepts and incorporates bulk cations [12,19,30] (Fig. 6). The ion first arrives to the channel entrance while the channel cavity is occupied by water molecule. Then, the positions of the water and the ion are exchanged. The very first cation provides visible stabilization for the stem and the stem is capable to re-shuffle the ion towards the center. Thus, the lifetime of a G-DNA stem with a vacant channel could be 1–10 ns. In one recent simulation the stem disintegrated after the ion capture, so more data would be needed to have statistics [27].

In summary, the G-DNA stems are never left vacant by cations and their stability in water results from cations associated with the tetrads. These qualitative results are assumed to be unaffected by the force field approximations, since they stem from balance of well-described nucleobase interactions and electrostatics. Vacant G-DNA stems in solution suggested recently by some ESI-MS experiments (see Section 2.16) are in a striking disagreement with theoretical data.

### 3.3 GCGC quartets, inosine and 6-thioguanine

Mixed quadruplexes with GCGC quartets were studied by NMR [109–111]. In simulations [12], the GCGC quartets adopt two distinct conformations, termed “closed” and “sheared” in good agreement with NMR. Stability of mixed quadruplexes is due to the cation-stabilized guanine quartets while a stem formed exclusively by GCGC quartets is unstable. G-DNA like quadruplexes lacking the G-quartets should not exist because of their genuine instability.

Inosine is capable to form cation-stabilized all-inosine quadruplex stem that is primarily stabilized by the quartet–cation interactions, which are very similar for inosine and guanine due to the similarity of their electronic structures (orientation of dipole moments) [38]. However, the inosine quadruplex (in contrast to the guanine quadruplex) with a vacant channel disintegrates within the first nanosecond of simulation having no time to catch any cation [14]. In absence of ions, the lack of the amino group is immediately visible. The difference between guanine and inosine H-bonding capability may affect the folding process of the quadruplexes by destabilizing intermediates that rely on base pairing (see Section 3.4).

Thioguanine destabilizes the quadruplex structure [112]. Although a single thioguanine is tolerated in simulations [13,14], its presence in G-DNA is penalized by the free energy difference of ∼4.4 kcal/mol. All-thioguanine quartet is visibly stressed while full thioguanine stem collapses immediately. The thiogroup has favorable interaction with Na^+^ cations and in addition is rather poorly hydrated, so its burial inside the structure could be convenient. However, this group is just too bulky for G-DNA.

### 3.4 Quadruplex stem formation—insight beyond the experiments

Formation of G-DNA structures is a slow process with a complex kinetics [113,114]. Most basic questions remain unclear, such as at what stage are the monovalent cations integrated into the structure and what is the atomic-level structure of the intermediates?

**Fig. 7:**
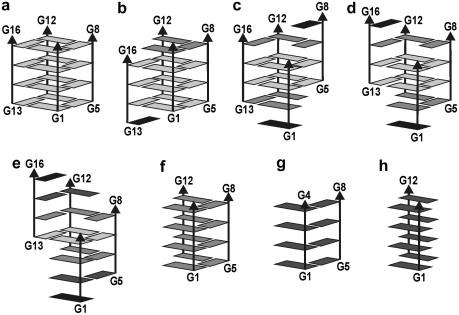
Starting structures of investigated intermediates [15]: (a) native stem, (b) stem with one strand shifted, (c) and (d) stem with two adjacent strands shifted down and up, (e) spiral stem, (f) triplex, (g) parallel edge duplex, and (h) parallel diagonal duplex.

Parallel d(G_4_)_4_ quadruplex was selected as the model system for computational analysis [15]. Various four-stranded, three-stranded and duplex assemblies were designed (Fig. 7) and simulated. Stable structures were suggested as possible intermediates of the quadruplex formation. Other structures that disintegrate on the ns-scale are unlikely to be involved in G-DNA formation.

**Fig. 8:**
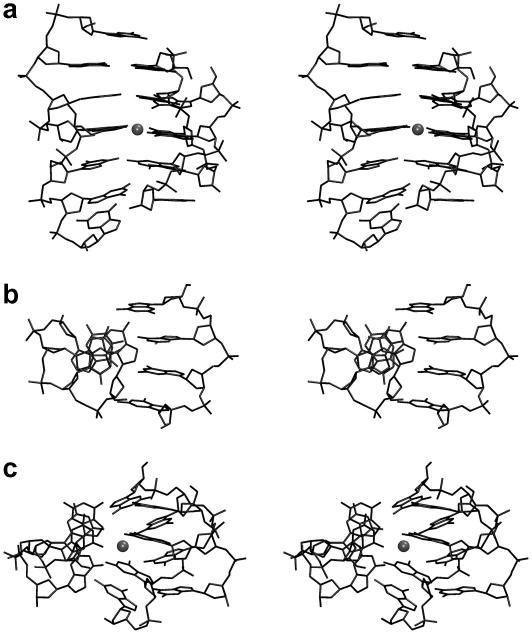
Stereo view of averaged structures of key simulated intermediates [15]: (a) spiral stem with one cation coordinated in the guanine quartet, (b) “cross-like” duplex and (c) “coil-like” triplex.

**Fig. 9:**
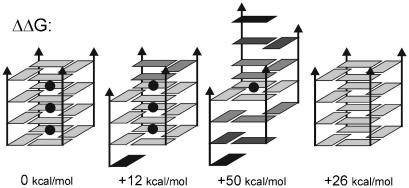
Relative MM-PBSA free energies. From the left to the right: native stem, slipped stem, spiral stem and vacant stem. Data adapted from Ref. [15].

Possible intermediates are four-stranded structures with mutually slipped strands with a reduced number of guanine quartets. “Spiral stem” is a structure with each strand shifted by one layer with only one guanine quartet in the center. The structure is stable on the ns-scale (Fig. 8a), is stabilized by one ion associated with the central quartet while the outer parts of the structure are stabilized by guanine self-pairing (here the guanine to inosine substitution would matter). In elevated temperature simulation the spiral rearranges towards the native G-DNA stem. Thus, later stages of quadruplex stem formation may involve a set of slipped structures with progressive reduction of the strand slippage. The final rearrangements from 3 to 4 quartets may be quite slow since cation-stabilized intermediates with several quartets are assumed to be already rather stable and rigid. The final slip could occur during the exchange of cations between solute and bulk solvent. Fig. 9 summarizes relative (quite approximate) MM-PBSA free energies for selected four-stranded G-DNA structures [15]. The native G-DNA stabilized by cations represents the free energy minimum and any strand slippage leads to the free energy increase. In comparison with the native G-DNA, the vacant quadruplex is significantly destabilized.

Neither “edge” nor “diagonal” straight d(GGGG)_2_ duplexes are stable but “cross-like” d(GGGG)_2_ structures (Fig. 8b) formed by two perpendicular strands with non-planar interbase hydrogen bonding could be suitable early stage intermediates. The “cross-like” structures are not stabilized by ions. Straight three-stranded structure (G-DNA stem with one strand removed) disintegrates swiftly but there is a stable d(GGGG)_3_ “coil-like” structure (Fig. 8c) formed by the above “cross-like” duplex interacting with the third strand via a cation.

The simulations allow hypothesize about the following single molecule scenario of formation of four-quartet parallel-stranded stems. The initial intermediates could involve the basepair-stabilized cross-like duplexes. Such structures can further associate in three- or four-stranded “coils” still primarily relying on base–base interactions but already involving transiently associated ions. The next step could be formation of partly structured stems. Once the first tetrad occurs it would be immediately stabilized by a cation. Such structure could be capable to step by step (by reducing the strand slippage) convert to the native stem. Long-lived intermediates might have been captured in recent FRET experiments though the experiments give no structural insight and likely do not capture all intermediates [115].

### 3.5 Guanine quadruplex loops—when simulations fail

Modeling of single-stranded loops is challenging due to (i) inherent difficulty to mimic the backbone electronic structure, (ii) substantial exposure of the loops to the solvent which increases the requirements for proper balance of solute–solvent interactions, (iii) competing substates and (iv) interactions of loops with cations.

Standard simulations starting from the experimental diagonal loop geometries of d(G_4_T_4_G_4_)_2_ were stable [16]. However, the ion binding site at the stem-loop junction was lost within 1–3 ns. Subsequent LES (see Section 2.12) simulations reproducibly localized the global minimum of the loops [16]. Unfortunately, the predicted geometry is very different from the experimental one obtained independently by NMR and X-ray [4,61,62] with variable ions and crystal packing (all experimental geometries are very similar). MM-PBSA shows that the LES-predicted topologies have the lowest free energies, so that LES and MM-PBSA calculations are mutually consistent. Thus, it was suggested that the parm94/99 force field predicts incorrect loop topology as the free energy minimum. Preliminary simulations of the parallel-stranded human telomeric quadruplex with groove loops (Fig. 3b) (unpublished data) are equally unsatisfactory, and the loops deviate from the experimental geometries even during conventional simulations. Hazel et al. carried out extended loop simulations [20,21] but in light of our results these structures could also be biased although they could properly reflect crude geometrical constraints stemming from the loop length.

### 3.6 The i-DNA stacking lesson. Is the base stacking always attractive?

**Fig. 10:**
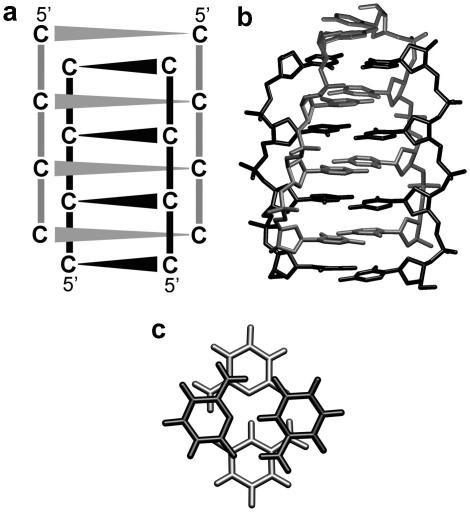
i-DNA (a) scheme, (b) 3D structure and (c) stacking of two hemiprotonated C–C^+^ base pairs in i-DNA.

Finally, let us comment on i-DNA tetraplex [116] (Fig. 10). The i-DNA stem consists of several consecutive protonated cytosine–cytosine homopairs. Such protonation stabilizes the planar base pairing. However, each base pair has charge of +1 which should be associated with enormous vertical electrostatic repulsion. The +1 charge is neatly redistributed over the cytosine rings, but the vertical *in vacuo* stacking between the consecutive base pairs is +25 to +30 kcal/mol, contrasting attractive stacking of −10 to −15 kcal/mol in B-DNA [117]. Despite this, fully protonated i-DNA stays beautifully stable in simulations [118]. The vertical repulsion between consecutive protonated base pairs in i-DNA is counterbalanced (over-corrected) by solvent screening, modulated by the overall topology of the i-DNA tetraplex. Thus i-DNA indeed has, in contrast to all other DNA conformational classes, repulsive intrinsic *in vacuo* stacking energy terms. The stability is due to common electrostatics, as the force field does not include any polarization, exocyclic group–aromatic ring interactions, resonance contributions, etc. Without computations, we would be left in a darkness regarding the origin of the vertical stabilization in i-DNA.

Strikingly contrasting is behavior of consecutive protonated cytosines in C^+^–G.C triples of Pyr-Pur.Pyr triplexes [119]. Consecutive protonated cytosines would be needed to recognize consecutive guanines in the second strand, resulting in arrangement of (C^+^–G)*_n_* basepair stacks very similar to the (C^+^–C)*_n_* basepair stacks in i-DNA. However, this sharply destabilizes the DNA triplex because the overall balance of forces results in less efficient screening of the vertical electrostatic repulsion compared with i-DNA. Thus sometimes we cannot easily transfer experience concerning molecular interactions between two DNA conformational classes. This underlines the complexity and promiscuity of molecular interactions in nucleic acids. Thus, advanced computations are often necessary to fully understand how the interactions really work.

## 4 Concluding remarks

Quadruplex simulation studies provide an instructive example of the successes and failures of contemporary MD studies of nucleic acids. When a qualitative task was properly formulated, the simulations were capable to provide unique insights into the properties of G-DNA and i-DNA stems. On the other side, attempts to carry out an in-depth study of the loop region of G-DNA molecules failed.
